# *Wolbachia*, *Sodalis* and trypanosome co-infections in natural populations of *Glossina austeni* and *Glossina pallidipes*

**DOI:** 10.1186/1756-3305-6-232

**Published:** 2013-08-08

**Authors:** Florence N Wamwiri, Uzma Alam, Paul C Thande, Emre Aksoy, Raphael M Ngure, Serap Aksoy, Johnson O Ouma, Grace A Murilla

**Affiliations:** 1 KARI-Trypanosomiasis Research Centre, P.O. Box 362-00902, Kikuyu, Kenya; 2 Yale University, School of Public Health, 60 College Street, 811 LEPH, New Haven, CT 06520, USA; 3 Department of Biochemistry and Molecular Biology, Egerton University, P.O. Box 536-20115, Egerton, Kenya

**Keywords:** *Glossina*, *Wolbachia*, *Sodalis*, Trypanosomes, Co-infection, Shimba Hills, Kenya

## Abstract

**Background:**

Tsetse flies harbor at least three bacterial symbionts: *Wigglesworthia glossinidia*, *Wolbachia pipientis* and *Sodalis glossinidius. Wigglesworthia* and *Sodalis* reside in the gut in close association with trypanosomes and may influence establishment and development of midgut parasite infections. *Wolbachia* has been shown to induce reproductive effects in infected tsetse. This study was conducted to determine the prevalence of these endosymbionts in natural populations of *G. austeni* and *G. pallidipes* and to assess the degree of concurrent infections with trypanosomes.

**Methods:**

Fly samples analyzed originated from Kenyan coastal forests (trapped in 2009–2011) and South African *G. austeni* collected in 2008. The age structure was estimated by standard methods. *G. austeni* (n=298) and *G. pallidipes* (n= 302) were analyzed for infection with *Wolbachia* and *Sodalis* using PCR. Trypanosome infection was determined either by microscopic examination of dissected organs or by PCR amplification.

**Results:**

Overall we observed that *G. pallidipes* females had a longer lifespan (70 d) than *G. austeni* (54 d) in natural populations. *Wolbachia* infections were present in all *G. austeni* flies analysed, while in contrast, this symbiont was absent from *G. pallidipes*. The density of *Wolbachia* infections in the Kenyan *G. austeni* population was higher than that observed in South African flies. The infection prevalence of *Sodalis* ranged from 3.7% in *G. austeni* to about 16% in *G. pallidipes*. Microscopic examination of midguts revealed an overall trypanosome infection prevalence of 6% (n = 235) and 5% (n = 552), while evaluation with ITS1 primers indicated a prevalence of about 13% (n = 296) and 10% (n = 302) in *G. austeni* and *G. pallidipes,* respectively. The majority of infections (46%) were with *T. congolense.* Co-infection with all three organisms was observed at 1% and 3.3% in *G. austeni* and *G. pallidipes,* respectively*.* Eleven out of the thirteen (85%) co-infected flies harboured *T. congolense* and *T. simiae* parasites. While the association between trypanosomes and *Sodalis* infection was statistically significant in *G. pallidipes* (P = 0.0127), the number of co-infected flies was too few for a definite conclusion.

**Conclusions:**

The tsetse populations analyzed differed in the prevalence of symbionts, despite being sympatric and therefore exposed to identical environmental factors. The density of infections with *Wolbachia* also differed between *G. austeni* populations. There were too few natural co-infections detected with the *Sodalis* and trypanosomes to suggest extensive inter-relations between these infections in natural populations. We discuss these findings in the context of potential symbiont-mediated control interventions to reduce parasite infections and/or fly populations.

## Background

Tsetse and trypanosomiasis continue to be a constraint to agricultural development in tsetse-infested regions of sub-Saharan Africa. Tsetse flies are the sole insect vectors responsible for transmission of both human and animal trypanosomiasis. The methods to control disease in the mammalian host are very limited. Traditional methods of vector control, such as traps and targets, have time and again proven unsustainable [1], turning the attention of scientists to the development of alternative methods, including genetic-based control such as the Sterile Insect Technique (SIT) and Incompatible Insect Technique (IIT).

All tsetse species are susceptible to trypanosome infections. However a differential vectorial capacity closely tied to differences in refractoriness to trypanosome infections are observed in tsetse [2,3]. In general, the *palpalis* group of flies tends to be poorer vectors of trypanosomes than the *morsitans* group. Although, the fly immune system is a key factor in this refractoriness [4], the natural symbiont-tsetse-trypanosome tripartite relationship has also been suggested to influence tsetse’s refractoriness. The presence of gut endosymbionts in *Glossina* and other obligate feeders has long been established [5]. All tsetse flies rely on the obligate primary symbiont *Wigglesworthia glossinidia* for vital physiological functions, including maintenance of fecundity and immunity [6,7]. In addition, some natural tsetse populations harbor the symbionts *Wolbachia* and *Sodalis.* While *Wolbachia* resides in reproductive organs, *Sodalis* is found in the tsetse midgut, which is also the site of trypanosome maturation. Thus, the interactions between symbionts and trypanosomes may provide an avenue for disease control.

The prevalence of symbiotic infections in different tsetse species and natural populations vary and indeed some individuals carry none except for the primary symbiont *Wigglesworthia*[8]. Recent work carried out with *G. f. fuscipes* from various populations in Uganda did not detect any *Sodalis* infections, but low density infections with *Wolbachia* were reported [9]. In the *G. p. palpalis* populations in West Africa, a low but statistically significant association was found between the presence of *Sodalis* and trypanosome infections [10]. However, the role of specific *Sodalis* genotypes in influencing susceptibility of the flies to infection by trypanosomes remains to be determined [11]. It appears that the infection prevalence of trypanosomes in natural populations may be influenced by the presence of different symbiotic microfauna the flies carry (reviewed by Weiss *et al*. [12,13]).

It has also been postulated that manipulation of the tsetse-endosymbiont interactions could present the next avenue to disease and vector control. Paratransgenic approaches, which genetically modify the symbiont in the vector, have proved successful in reducing the capacity of triatomine bugs to transmit the *T. cruzi* parasite [14]. The viability of this approach has also been demonstrated in sandflies for the control of *Leishmania*[15], and in mosquitoes for malaria control [16-18]. The IIT method is based on the cytoplasmic incompatibility (CI) phenotype expressed by some strains of *Wolbachia*. CI expression results in embryonic lethality when *Wolbachia* infected males mate with un-infected females (referred to as uni-directional CI). Bi-directional CI is expressed when individuals with different incompatible strains of *Wolbachia* mate. The CI effect of *Wolbachia* infections has been exploited in semi-field trials to control the mosquito *Culex pipiens*[19], and the medfly *Ceratitis capitata*[20]. Recently, CI was experimentally demonstrated to be also expressed in *G. m. morsitans*[21]. In addition to CI, *Wolbachia* infections have been shown to confer additional host-fitness effects, which can be applied for disease or vector control. Some strains of *Wolbachia* have been shown to decrease the lifespan of their hosts [22,23], or decrease the susceptibility of their host to transmit parasitic [24] and viral infections [25]. Therefore, it is clear that novel alternative vector control methods based on use of microfauna exist and some have passed the proof of concept, being at field-testing stage [26]. For successful translation of laboratory results in actual field applications, an in-depth understanding of the prevailing natural field dynamics is required. Data on natural infection frequency and information on the circulating symbiont strain types is critical in order to evaluate the potential use of endosymbionts to modify insect vector population traits [27].

There is limited information available on *Wolbachia* and *Sodalis* infections in Kenyan tsetse populations, often describing a single collection [8,28]. The objective of the present study was to perform a temporal analysis for infection prevalence of the symbionts *Wolbachia* and *Sodalis* in the coastal tsetse belt of Kenya where two species, *G. austeni* and *G. pallidipes* exist in sympatry. We also compared the Kenyan *G. austeni* populations to two other populations obtained from South Africa. We investigated the age structure of the two populations as well as trypanosome infection prevalence over three years in order to detect temporal variations. The over-arching aim of this study was to build the foundation on tsetse’s microbial partners, which can be harnessed to develop genetic based methods to reduce trypanosome infections.

## Methods

### Study area

**Figure 1:**
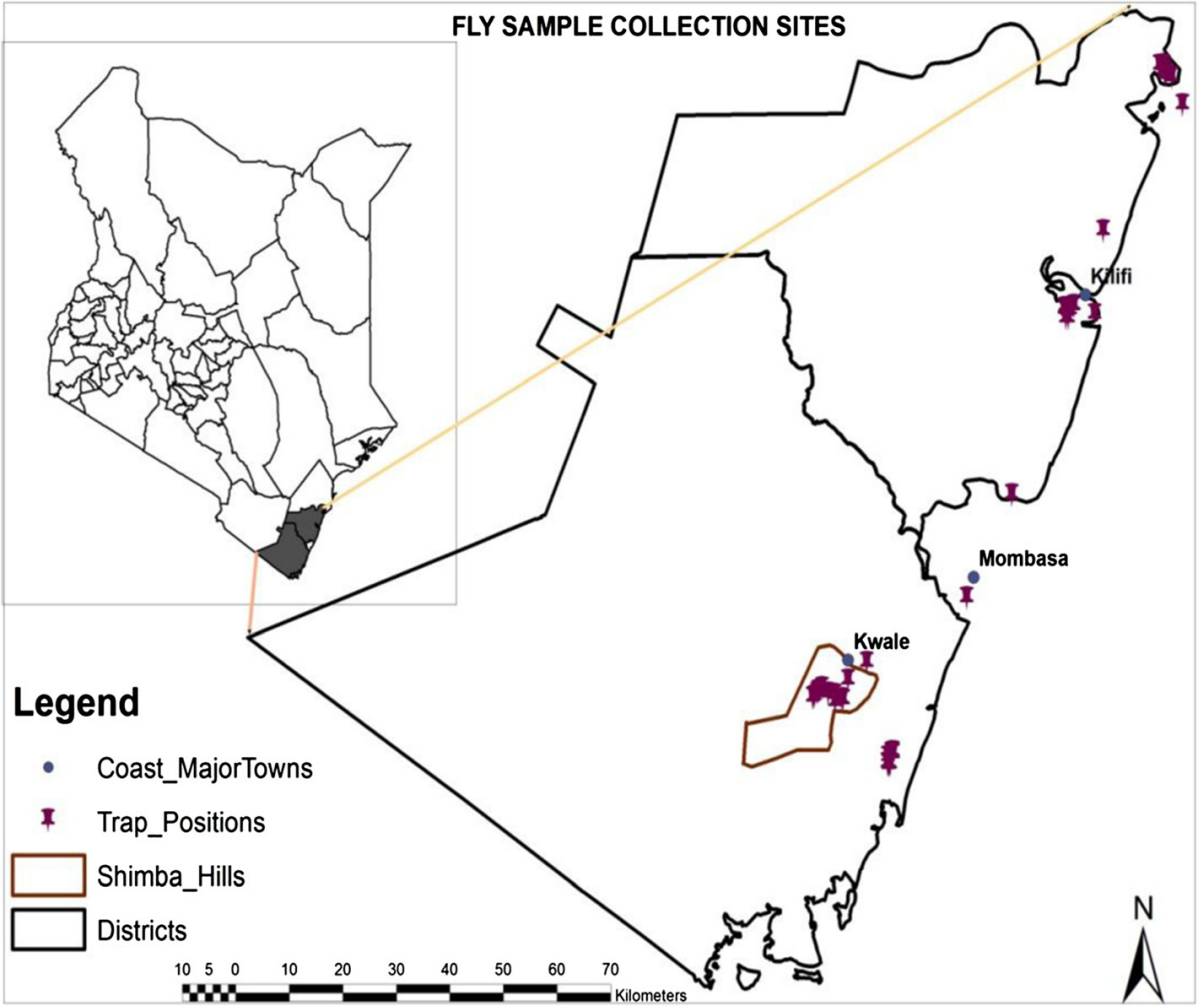
Fly sample collection sites in Shimba Hills, Kwale and Arabuko-Sokoke, Kilifi.

Tsetse flies were trapped from two national reserves in the coastal region of Kenya, namely Shimba Hills National Reserve (SHNR) in Kwale District and the Arabuko-Sokoke National Reserve (ASNR) in Malindi District between 2009–2011 (Figure 1). Both areas are located in the coastal lowlands agro-ecological zones 2–4 which are characterized by sub-humid conditions. SHNR lies between latitude 4°20′S and longitude 39°31′E, at an altitude of 120–450 m and covers an area of about 250 km^2^. The area has temperatures ranging between 24–36°C and rainfall averages 900–1200 mm per annum, falling in the short rains of October-December and the long rains of March-June. A wide range of wild animals can be found here including elephants, buffalo, antelope and waterbuck. The ASNR is located at latitude 3°16′S and longitude 39°49′E. The park itself covers an area of about 6 km^2^ and is part of the larger Arabuko-Sokoke forest, the largest remaining fragment of coastal forest in East Africa. The forest contains three forest types namely mixed forest, *Brachystegia* and *Cynometra* tree species, each of which protects different communities of plants and animals. Like the SHNR, a wide variety of wild game is present including mongoose, duikers, forest elephants, African civets, baboons and Vervet monkeys. There are three species of tsetse flies found in the study area, namely *G. pallidipes*, *G. austeni* and *G. brevipalpis*.

### Fly collections

*Glossina austeni* and *G. pallidipes* were trapped using either standard biconical trap [29], the Ngu2G trap [30] or the sticky mono-panel trap [31] whose lower half was coated with a thin layer of Temoocid® adhesive (Kollant SPA, Italy). The traps were baited with a plastic sachet containing 1 ml each of 4-methylphenol, 3-*n*-propyl phenol and 1-octen-3-ol. In addition, a soda bottle containing acetone was placed next to the trap. All the odours were dispensed by placing them on the windward side [32]. The traps were left in place for between 6–24 hours depending on the tsetse density in the particular area. All trap sites were geo-referenced using Garmin GPSmap76 (Garmin Corporation, Olathe, KS, USA). During the study period, all fly collections in Kenya were conducted during the months of October-November, coinciding with the short rainy season. On collection, fly catches from each trap were sorted by species and a proportion of them were preserved in 50 ml Falcon tubes containing absolute ethanol while the remainder were dissected for parasite detection. Records of total catches, sex, date and area of collection were maintained for cross-referencing purposes. A comparative sample of *G. austeni* (n = 74) collected from south of Lake St. Lucia, South Africa (LSLSA) and from the northern part of the lake, specifically Mbazwana, Muzi, Phinda and Tembe (“northern”) was also analyzed.

### DNA extraction

Upon removal from ethanol storage, individual flies were blotted dry on a paper towel and air-dried overnight at room temperature. Total genomic DNA was extracted from individual whole fly samples (minus legs and wings) or individual fly sections/organs (head, thorax, abdomen, legs) using either the DNeasy® Blood and Tissue Kit (Qiagen Sciences, MD, USA) according to the manufacturer’s instructions or by using the salting out protocol [33]. For *G. austeni* 155 and 141 samples were extracted by commercial kit and salt precipitation respectively, whereas for *G. pallidipes* these were 104 and 197 samples respectively. For the latter procedure, flies were crushed in 300 μl TNES buffer (50 mM Tris pH 7.5, 400 mM NaCl, 20 mM EDTA, 0.5% SDS) and incubated for 3 hours at 56°C in the presence of proteinase K (20 mg/ml). 5 M NaCl was then added to the solution and centrifuged to precipitate the proteins. The supernatant was removed and cold ethanol added to it. A further centrifugation step to sediment the DNA was carried out followed by an additional wash in ethanol. The final DNA product was eluted in 30-50 μl of TE buffer (10 mM Tris–HCl pH 7.5, 1 mM EDTA) and stored at −20°C until further processing.

### *Wolbachia* and *Sodalis* screening

The presence of *Wolbachia* was determined by amplification of the *Wolbachia* surface protein (*wsp*) gene using the primers 81 F/691R [34,35], which produced a fragment of 600 bp and confirmed by a *Wolbachia-*specific 16S rRNA assay [36]. The presence of *Sodalis* was determined using the primers GPO1F/GPO1R [37]. PCR assays were performed using 1 μl of template DNA and standard conditions [9]. For each assay, a negative control (no DNA) as well as a positive control using DNA isolated from *Sodalis morsitans* was included. The quality of sample DNA was verified using insect-specific 12S mitochondrial markers [38]. After completion of the PCR run, 10 μl of the amplification product was analyzed by electrophoresis in TAE buffer on a 1–2% agarose gel together with a 100 bp DNA ladder size standard (Invitrogen, Carlsbad, CA, USA) and visualized using ethidium bromide.

### Determination of trypanosome infection in flies

In the field, where possible, a small proportion of the captured flies were dissected in PBS using the method of Lloyd and Johnson [39] and examined microscopically for presence of trypanosomes. Circulating trypanosome species were identified based on their location in the fly, such that infections in the proboscis only were categorized as *T. vivax,* infections in both proboscis and midgut as *T. congolense* and infections in proboscis, midgut and salivary glands as *T. brucei spp.* Infections found in the midgut only were identified as immature infections [39]. Trypanosome infection was also detected by PCR using universal primers ITS1 CF and ITS1 BR [40]. Following DNA extraction, PCR reaction was performed in 20 μl reactions for 35 cycles at an annealing temperature of 58°C. For each PCR run, DNA from an uninfected colony fly and from known trypanosome stocks were included as negative and positive controls, respectively. Electrophoresis and visualization was carried out as described above. Identification of the infecting trypanosome(s) was based on the product size as described by Njiru *et al*. [40].

### Age structure of the different populations

Fly age was estimated using representative samples collected over three years in SHNR where both *G. austeni* and *G. pallidipes* were present. Ageing was performed using wing fray analysis for males and ovarian ageing for females. The wings from 100 randomly selected male flies of both species were scored based on the degree of tearing on the trailing edge of the wing [41]. An additional subset of females was used to estimate the age of the population using the ovarian ageing technique [42].

## Results

### Tsetse fly density

A total of 6,384 *G. pallidipes,* 1,030 *G. austeni* and 504 *G. brevipalpis* were captured in 226 trap-days during the study period. Average trap catches varied with location, fly species and trap design. As an illustration, the sticky monopanel traps which were deployed 66 trap-days only in the first year of study captured 2.8 flies/trap/day (FTD) of *G. austeni* as compared to 0.9 and 0.5 FTD captured by the biconical and NGU traps respectively. However, considering all trap catches irrespective of trap type the FTDs over the three-year period was 24, 4.1 and 1.9 flies for *G. pallidipes, G. austeni* and *G. brevipalpis* respectively. All areas recorded lower FTDs in the year 2010 as compared to 2009 and 2011. Overall, the highest FTDs were recorded for *G. pallidipes* in SHNR with densities in excess of 60 FTD. No *G. pallidipes* flies were captured in the ASNR.

### Age structure of *G. austeni* and *G. pallidipes*

Our analysis revealed a relatively younger male *G. austeni* population than that of *G. pallidipes*. The mean age of male *G. austeni* was estimated at 18 days, while that of *G. pallidipes* was estimated at 30 days. In contrast, ovarian ageing indicated that females of both species had comparable longevity and there was no significant difference between the frequency distribution of females among the eight ovarian categories (Table 1).

**Table 1:** **Frequency distribution (%) of the ovarian categories in female *G. austeni *and *G. pallidipes *as determined by ovarian ageing**

**Ovarian category**	**Estimated age (days)**	**Frequency (%)**		**P-value**
		***G. pallidipes *(n = 103)**	***G. austeni *(n = 67)**	
0	0–8	1.6	4.5	0.6812
1	8–19	7.8	14.9	0.2011
2	20–30	30.1	25.4	0.6016
3	30–40	22.3	22.4	1.000
4	40–50	13.6	16.4	0.6607
5	50–60	5.8	3.0	0.4820
6	60–70	12.6	10.4	0.8090
7	70–80	4.9	3.0	0.7052

There were no significant differences in number of females per ovarian category between the two species.

### *Wolbachia* and *Sodalis* infection prevalence in the two tsetse species

**Figure 2:**
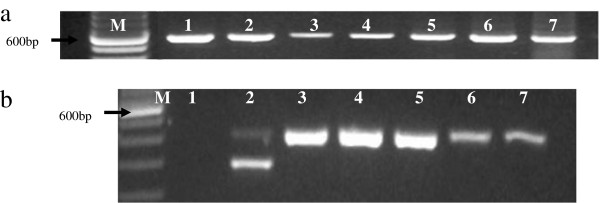
**PCR analysis of *Wolbachia *organ distribution and comparative inter-country density. (a)** Distribution of *Wolbachia* in *G. austeni* organs using *wsp*. The results of two representative flies are shown: M-100 bp DNA marker, lane 1-♂head, lane 2-♂thorax, lane 3-♂abdomen, lane 4-♀reproductive tract, lane 5-♀head, lane 6-♀thorax, lane 7-♀legs. Arrow indicates 600 bp. **(b)** 16S rRNA analysis of *G. austeni* from Kenya and South Africa: M-100 bpDNA marker, Lane 1-NC, Lane 2-*G. morsitans*, Lanes 3, 4, 5-*G. austeni* Kenya, Lanes 6, 7-*G. austeni* South Africa. Arrow indicates 600 bp. Results of five representative samples are shown here out of 50 samples analyzed.

The quality of template DNA, in particular the salt-extracted samples, was confirmed by the 12S assay as described above and negative samples excluded from further analysis. Subsequently, 370 *G. austeni* comprising 206 females and 164 males were analyzed for *Wolbachia* infection prevalence. Of these, 100 samples were collected from ASF, 196 from SHNR and the remaining 74 originated from South Africa. All 370 samples were *Wolbachia-*positive using the *wsp* gene. The presence of *Wolbachia* infections was confirmed in a subset of these samples (n = 50) using the 16S rRNA-based PCR assay, and all samples were positive. Using this assay, only one amplification product of ~400 bp was observed. This is in contrast to the profile obtained for *G. m. morsitans*, where an additional band of ~300 bp is observed. In the organ-specific assay, *Wolbachia* was detected all body sections assayed including head, thorax, abdomen and legs (Figure 2a). Comparing the Kenyan and the South African populations, the intensity of the PCR amplification products from the latter population was consistently weaker than those of the former population, despite using the same volume of total genomic of DNA as PCR template (Figure 2b). Out of 161 female and 141 male *G. pallidipes* from SHNR analysed, none was positive for *Wolbachia.*

The prevalence of *Sodalis* infections in *G. austeni* was 2% (n = 100) in ASF and 4.6% (n = 196) in SHNR as detailed in Table 2. The difference in prevalence was not significant (P = 0.3443) between the two areas. In the South African samples, the prevalence in LSLSA samples was 70.8% (n = 24), while only one out of 50 flies analysed from the “northern” population was *Sodalis* positive. The prevalence of *Sodalis* was significantly different inter-country (p < 0.0001). In the case of *G. pallidipes*, 16% (n = 302) were infected with *Sodalis* (Table 2). Infection was biased towards females in *G. austeni* (p < 0.0001), but no significant sex difference in infection was observed in *G. pallidipes* (P = 0.0577).

**Table 2:** ***Sodalis *prevalence in *G. austeni *and *G. pallidipes *from different locations**

**Species**	**Source**	***n***	**Number infected**		**Prevalence**
			**F**	**M**	
*G. austeni*	ASF, Kenya	100	2	0	2% (0.00243 ≤ p ≤ 0.07038)
	SHNR, Kenya	196	6	3	4.6% (0.02121 ≤ p ≤ 0.08538)
	St. Lucia, S. Africa	24	17	0	70.8% (0.48905 ≤ p ≤ 0.87385)
	northern, S. Africa	50	1	0	2% (0.00051 ≤ p ≤ 0.10647
*G. pallidipes*	SHNR, Kenya	302	32	16	15.9% (0.11957 ≤ p ≤ 0.20515

“northern” refers to sites north of LSLSA. F-females, M-males; prevalence % given with 95% confidence intervals in brackets.

### Temporal variation of *Sodalis* prevalence

There was no statistical difference between *Sodalis* infection in ASF and SHNR and therefore the data were pooled to compare the temporal variance in prevalence. The total mean% prevalence for *Sodalis* infection for the 3-year period was significantly higher in *G. pallidipes* than in *G. austeni* (*p* < 0.0001). There was no significant difference between the two species of flies in the year 2009 (P = 0.8014). However, as shown in Table 3, in 2010 and 2011 the prevalence was significantly higher in *G. pallidipes* than in *G. austeni* (p < 0.0001, Fisher’s exact test).

**Table 3:** **Temporal variation of % *Sodalis *infection prevalence in Kenyan *G. austeni *and *G. pallidipes***

	**Year**			
	**2009**	**2010**	**2011**	**Total**
*G. austeni*	8.6% (116)	1.5% (66)	0% (114)	3.7% (296)
*G. pallidipes*	7% (100)	27% (100)	13.7% (102)	15.9% (302)
*p-value*	*p* = 0.8014 (NS)	*p* < 0.0001	*p* < 0.0001	*p* < 0.0001

Numbers in brackets represent *n*. The difference in total infection prevalence between the two species was highly significant.

### Trypanosome infection in *G. austeni* and *G. pallidipes*

**Figure 3:**
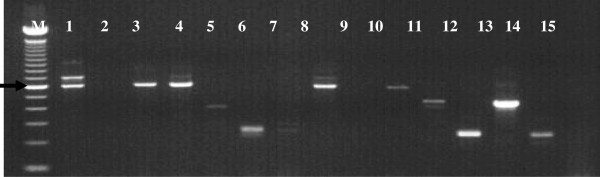
**Identification of trypanosomes infecting tsetse flies using ITS1.** Identification is based on product size. M-100 bp DNA marker: lanes 1, 3, 4, 8, 10-*T. congolense* sub-types, Lane 5-*T. simiae*: lanes 6, 7-*T. vivax*: lane 11-*T. brucei*: lane 13-positive control *T. brucei*: lanes 12, 14-positive controls for *T. vivax*. Lane 15-negative control. Arrow indicates 600 bp.

**Figure 4:**
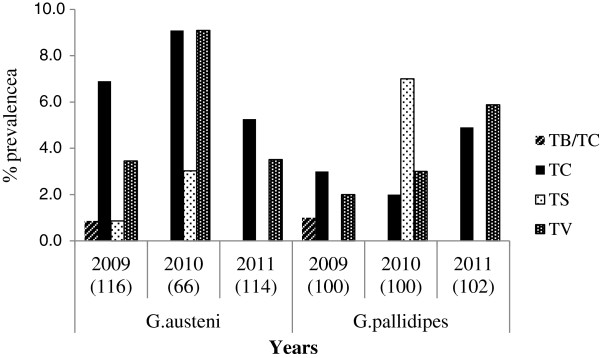
**Trypanosome species infecting *G. austeni *and *G. pallidipes *for the period 2009–2011 as determined by PCR.** Numbers in brackets represent total number of samples analysed for that specific year. TV-*T. vivax*, TS-*T. simiae*, TC-*T. congolense*, TB/TC-*T. b. brucei/T. congolense* mixed infection.

Dissection of 787 flies comprising 235 *G. austeni* and 552 *G. pallidipes* yielded infection rates of 6% and 4.9% respectively. PCR analysis detected 71 trypanosome infections with various trypanosomes from 598 and 74 flies from Kenya and South Africa respectively (Figure 3). In the Kenyan *G. austeni* population, trypanosome prevalence in ASF was 13% (n = 100) while in SHNR, it was 12.7% (n = 196). However, the difference in prevalence was not significant (P = 1.0000). In the South African population, no trypanosomes were detected in the LSLSA population, while a prevalence of 8% was detected in the northern population. An infection prevalence of 9.6% (n = 302) was observed in *G. pallidipes*. Combining all the species- and country-specific data, the total trypanosome prevalence was significantly higher in the Kenyan samples (P = 0.0425), but no significant difference was observed between the two fly species (P = 0.2435). In both tsetse species, proportionately more females than males were infected. This difference was significant in *G. pallidipes* (p < 0.0001) but not in *G. austeni* (P = 0.8694). A temporal effect was evident with both species having a higher parasite infection rate in 2010 (Figure 4). Chi-square test showed that the differences in prevalence rates for the three years was significant in both *G. austeni* (χ^2^ = 6.812, df = 2, P = 0.00332) and *G. pallidipes* (χ^2^ = 2.321,df = 2, P = 0.3133). ITS1 primers identified that several species of trypanosomes infect tsetse flies (n = 71) in the study area (Figure 4), the most common of which were *T. congolense* (46.5%), *T. vivax* (38%) and *T. simiae* (14.1%). A small proportion of flies (2.8%) had mixed infections of *T. b. brucei* and *T. congolense* (Table 4).

**Table 4:** **Trypanosome species detected in *G. pallidipes *and *G. austeni. *Trypanosome species detected in *G. pallidipes *and *G. austeni***

**Species**	***Source***	***n***	***T.c.***	***T.v.***	***T.sm.***	***Tbb/Tc***	**Total**
*G. austeni*	ASF, Kenya	100	8	3	1	1	13 (13%)
	SHNR, Kenya	196	12	11	2	0	25 (12.7%)
	St. Lucia, S. Africa	24	0	0	0	0	0/24 (0%)
	northern, S. Africa	50	2	2	0	0	4/50 (8%)
*G. pallidipes*	SHNR, Kenya	302	11	11	7	1	29 (9.6%)

*T.c*.-*T. congolense*, *T.v*.-*T. vivax, T. sm.*-*T. simiae, T.b.b.*-*T. b. brucei.* “northern” refers to sites north of LSLSA.

### Co-infection of *Sodalis* and trypanosomes

Only a small proportion of flies sampled harboured both trypanosomes and *Sodalis*. Of these 3.3% (n = 302) and 1% (n = 296) were found in *G. pallidipes* and *G. austeni,* respectively. Flies that were microscopically-positive for trypanosomes were not always infected with *Sodalis* bacteria. The association between trypanosomes and *Sodalis* infection was statistically significant in *G. pallidipes,* but not in *G. austeni* (Table 5). Of the 13 co-infected flies, 5 were infected with *T. congolense*, 6 with *T. simiae* and 2 with *T. vivax.*

**Table 5:** ***Sodalis *and trypanosome co-infection in field collected *G. pallidipes *and *G. austeni***

	***G. pallidipes *(n = 302)**		***G. austeni *(n = 296)**	
	**T+**	**T-**	**T+**	**T-**
S+	10 (3.3%)	38 (12.6%)	3 (1.0%)	8 (2.7%)
S-	19 (6.3%)	235 (77.8%)	35 (11.8%)	250 (84.5%)
*P*-value	P = 0.0127 *		P = 0.1554 (NS)	

* significant, NS-not significant, S^+^/S^-^*Sodalis* positive/negative, T^+^/T^-^ trypanosome positive/negative.

## Discussion

### Fly catches

Three species of tsetse flies were captured in SHNR in increasing order of *G. brevipalpis*, *G. austeni* and *G. pallidipes.* The mixed type of vegetation found in this area provides suitable habitat for these savannah-dwelling species. About 75% of the total catch was *G. pallidipes*, influenced in part by the sampling tools. The study used biconical and Ngu traps, which are more effective for the capture of *G. pallidipes* than of *G. austeni*[43]*.* Sticky mono-panel traps are recommended for sampling of *G. austeni*[31] and indeed in the present study, this trap design captured more *G. austeni* than the other two traps. However, trap comparison was not an objective of our study and we did not therefore perform idealised trap comparison experiments. Flies caught on the sticky monopanel traps proved difficult to handle thereafter during dissection or DNA extraction due to the adhesive used on the panels and thus this trap was not routinely used. No *G. pallidipes* flies were captured in Arabuko-Sokoke forest. This can be attributed to the observation that *G. pallidipes* prefers open savannah grassland habitats, whereas the ASF habitat is composed mainly of dense indigenous forest.

### Prevalence of *Wolbachia* in *G. austeni* and *G. pallidipes*

All *G. austeni* individuals analyzed were shown to be infected with *Wolbachia*. This result indicates an increase in infection prevalence towards complete fixation of *Wolbachia* considering that available 1996 data reported a prevalence of 48% in Shimba Hills and 98% in South Africa, respectively [28]. Population genetics models using data derived from *G. m. morsitans* predict that it would take about 18 months for *Wolbachia* prevalence to reach 95% of the fixation prevalence starting with a release ratio of 10% *Wolbachia* infected flies [21]. In relation to vector control, *Wolbachia*-mediated cytoplasmic incompatibility (CI) is often proposed as a possible control intervention [44] and recently, uni-directional CI has been demonstrated to occur in a *G. morsitans* laboratory line [21]. Although the presence of CI in *G. austeni* remains to be confirmed, the high infection prevalence observed in this study indicates that strong CI may be operating in this species as well. Our future work will confirm this by cross-mating natural populations with heterogeneous *Wolbachia* infection or by using tetracycline-cured laboratory lines.

The *Wolbachia* 16S rRNA analysis revealed only one band in *G. austeni*, unlike in *G. morsitans,* which displayed two distinct bands under the same amplification conditions. In the latter species, the larger product (~400 bp) represents the cytoplasmic product located in the reproductive organs, and which can be eliminated by tetracycline treatment. The smaller 300 bp product represents either the *Wolbachia* infection found in various tissues or is a degenerate 16S rRNA copy suggestive of the lateral transfer of this gene from *Wolbachia* into *G. morsitans*[36]. Our results augment the available information highlighting the differences between *Wolbachia* strains in these two species and which has been confirmed by MLST analysis [36]. Our findings based on the *Wolbachia* 16S rRNA gene do not however, unequivocally confirm the presence or absence of an LGT event in *G. austeni*. It is therefore possible that the *Wolbachia* fixation we observed in *G. austeni* may be due to a LGT event, rather than an authentic *Wolbachia* infection. While it would be possible to confirm this using tetracycline-treated laboratory lines, this facility was not available to us during the period of the study. Additional work is therefore required to ascertain the presence of *Wolbachia* in aposymbiotic *G. austeni*.

*Wolbachia* density was greater in the Kenyan samples than in the South African samples based on the intensity of the PCR amplification product. The bacterial density and subsequently, the strength of CI expression may be influenced by the infecting *Wolbachia* strains. [45] However, since *Wolbachia* strains infecting the Kenyan and the South African tsetse populations are virtually identical [36], strain differences do not adequately address this discrepancy. Average *Wolbachia* densities may also vary between populations of the same species as a consequence of environmental factors [46], and it is possible that the density difference observed in the present study is population-specific. *Wolbachia* density levels in the different populations representing the *G. austeni* belt stretching many kilometers along the East African coast, would be of interest to explore. Also of interest would be the host genetic differentiation and the presence and levels of CI expression in these populations.

No *Wolbachia* infection was found in the *G. pallidipes* populations analyzed*.* This result is consistent with previously reported zero infection rates in Kenyan and Ugandan *G. pallidipes* populations [28]*.* Recently, however, a prevalence of 1.2% was reported from 1,823 samples originating from Ethiopia, Tanzania, Zambia and Zimbabwe [36]. It is likely that *G. pallidipes Wolbachia* infection is either absent or of too low a density to be detected using the standard PCR assay. *Wolbachia* infection in *G. f. fuscipes* which were undetectable by standard *wsp* PCR analysis were subsequently detected using Southern blot PCR technique [47,48] and sequential amplifications [9]. The density of *Wolbachia* may play a role in expression of CI [49], thus the physiological significance of low-density *Wolbachia* infections in nature needs to be analyzed. *G. pallidipes* is a major vector for both human and animal trypanosomiasis in Kenya, and has been the target species in previous control programmes established in Nguruman, Busia and Lambwe Valley. For *Wolbachia*-based control strategies to be applied, a method of introducing the bacteria into this species would have to be developed. Tsetse are viviparous and the developing larvae acquire *Wolbachia* through germ-line transmission. Although methods for propagating *Wolbachia in vitro* are available [50], it may be difficult to introduce *Wolbachia* into the developing eggs because the embryonic microinjection technique used in other arthropods [51] cannot be easily applied in tsetse. The difficulty of mass-rearing this species [52], in order for the establishment of a sufficiently large self-sustaining laboratory colony of *Wolbachia*-infected *G. pallidipes* for large scale intervention would need to be overcome.

### Prevalence of *Sodalis* in *G. austeni* and *G. pallidipes*

Most commensal symbionts are considered non-essential and they are generally found at intermediate frequencies within host populations [53]. *Sodalis* was detected in Kenyan *G. austeni* and *G. pallidipes* at 3.7% (n = 296) and 15.9% (n = 302) prevalence respectively. In contrast, *Sodalis* prevalence in the LSLSA *G. austeni* population was 70.5%. This difference could be occasioned by environmental factors, which have been shown to influence symbiont densities in laboratory experiments [54]. In the present study, higher prevalence was obtained in fly samples from trap sites that are closer to the coastline (LSLSA) than those further inland, which may be attributed to marine-induced differences in the micro-climate. *Sodalis* infection prevalence also showed variation among the different species, with higher *Sodalis* infections in *G. pallidipes*. Among the riverine species, although *G. f. fuscipes* in the Lake Victoria, region apparently lack *Sodalis* infections [8,9], the prevalence in west African *G. p. palpalis* has been reported to range from 9.3% to 54.9% [10,37,55].

### Temporal variation of *Sodalis* infection in *G. pallidipes* and *G. austeni*

In the present study, the prevalence of *Sodalis* infection in both species more than doubled in 2010 as compared to the previous year. Temporal variations in symbiont infection prevalence may be correlated with climatic and ecological factors. In weevils, higher infections with *Sodalis*-allied symbiont (*Sitophilus oryzae* primary endosymbiont (SOPE), *Wolbachia* and *Rickettsia* have been reported at localities of higher temperature [56]. Weather data from the Kenya coast indicates that the year 2010 was characterised by a poor short rainy season (October-December) when the depressed rainfall was coupled with higher temperatures as the dry weather conditions extended into the traditional short rainy season [57]. The samples analysed in this study were collected during the months of October-November when hotter, drier conditions prevailed. In contrast both 2009 and 2011 were characterized by near-normal weather conditions. We postulate that environmental factors may have had an effect on the *Sodalis* prevalence in *G. austeni* and *G. pallidipes*

### Age structure of the sympatric *G. austeni* and *G. pallidipes*

*G. austeni* males in the SHNR have a predicted shorter lifespan than the sympatric *G. pallidipes,* whereas the longevity of females is comparable in the two species*.* Some strains of *Wolbachia* have a life-shortening effect on their hosts. In a laboratory study, the *wMel*Pop-CLA strain was shown to halve the lifespan of *A. aegypti*[22]*.* It has been hypothesized that this strain may be similar to that found in *G. austeni*[58]. Against this background, and considering the absence of *Wolbachia* in the sympatric *G. pallidipes* population, it could be possible that *Wolbachia* infection could be one of the factors contributing to shorter lifespan in *G. austeni.* However, the observation of this phenomenon in only male *G. austeni* may not be fully explained by this hypothesis. Future research will work towards confirming this finding in the field.

### Trypanosome infection in tsetse flies

The observed trypanosome infection rates of 12.8% and 9.6% for *G. austeni* and *G. pallidipes* respectively are comparable with reported infection rates of 8.7% in *G. pallidipes* from the same area [59]. Our finding of *T. congolense* as the most common trypanosome circulating in this area is also consistent with previous reports of high cattle infection rates (upto 18%), the majority of which were *T. congolense* infections [60]. Trypanosome infection prevalence was consistently higher in *G. austeni* than in *G. pallidipes.* It has been noted that despite the lower apparent density of *G. austeni* relative to other sympatric species, *G. austeni* often harbours higher levels of trypanosome infection [61]. The results obtained in the present study are in contrast to those obtained from Rufiji district along the Tanzanian coast whereby *G. pallidipes* was found to have a much higher infection prevalence than *G. austeni*[62]. Notably, more than half of the flies analyzed from the Rufiji population were infected with between two to five trypanosomes species while in this study, mixed infections accounted for only 2.8% of all infections. It is inferred that the presence of a wide spectrum of potential tsetse hosts, as well as sequential feeding upon multiple hosts may contribute to an abundance of mixed trypanosome infections [63]. However, since both of these regions have a wide variety of wild animals, the difference in prevalence of mixed infections may be a reflection of fly immunity, parasite maturation factors and possibly varying microfauna [64].

### Co-infection with *Sodalis* and trypanosomes

*Sodalis* is transmitted through the milk gland secretions of the mother to the intra-uterine developing larva [65], and is therefore present in the gut at eclosion in the newly emerged teneral fly. On the other hand, trypanosome infection is acquired later in the presence of an infected blood meal source. The establishment of trypanosomes involves complex interactions and various factors including lectin levels in the tsetse fly gut at the time of parasite uptake, fly species, sex, age and symbiotic associations in the tsetse fly gut play a part in determining the success or failure of parasite establishment [2]. Midgut symbionts such as *Sodalis* may exhibit a chitinolytic activity [66] which increases the susceptibility of tsetse fly to trypanosome infection [11]. Of the almost 600 tsetse samples that we analyzed, less than 2% were found co-infected with both *Sodalis* and trypanosomes. This result is not surprising given that very few flies were positive for any of the two organisms. Only ten *G. pallidipes* and 3 *G. austeni* were co-infected with both microorganisms. Therefore, despite positive correlation between presence of *Sodalis* and midgut trypanosome infections in natural populations [11,67], it is clear that tsetse flies can become infected with trypanosomes readily in the absence of *Sodalis* infections both in the laboratory and in the field [7,68]. The low co-infection rates observed in the present study are in contrast with the rates of 32.2% reported in *G. p. palpalis*[10]. It is, however, worth noting that in both the present study and the *G. p. palpalis* study, the trypanosomes species infecting *Sodalis*-positive flies belong mainly to the sub-groups *Trypanozoon* and *Nannomonas*. Intercommunity effects have been observed between symbionts and pathogens, where a negative correlation between *Wolbachia* and trypanosomes and a positive correlation between the salivary gland hypertrophy (SGH) virus and trypanosomes were noted in Ugandan *G. f. fuscipes*[9]. Thus, it remains to be seen if the apparent absence of *Wolbachia* infections in *G. pallidipes* may render these populations more susceptible to host *Sodalis* and/or trypanosome infections. Further, more work is required to determine whether the synergistic effect of *Sodalis* on trypanosome establishment and maturation may apply favorably to trypanosome species that particularly involve the tsetse midgut stages [69]. However, in this study the number of co-infected flies was too low to provide a strong argument for the antithesis. Further work on this aspect will serve to provide conclusive evidence.

## Conclusion

In summary, this study found that the natural prevalence of *Sodalis* and *Wolbachia* symbionts in *G. pallidipes* and *G. austeni* in coastal Kenya differ between the two species. The apparently absent or possibly low-titer infections observed in *G. pallidipes* complicate the feasibility for application of a *Wolbachia*-mediated CI control approach against this species. However, IIT could be possible for *G. austeni* if the CI effect is confirmed in this species. In the populations analysed in East Africa, there is low level of natural co-infection with the secondary endosymbiont and trypanosomes and this could indicate limited influence of *Sodalis* on trypanosome infection. Proposed symbiont-mediated control interventions should take these differences into account and particularly the low levels of infection.

## Competing interests

The authors declare that they have no competing interests.

## Authors’ contributions

FNW, RMN, SA conceived the experiments, FNW, PTC collected the field samples, JOO, SA contributed reagents, FNW, UA, EA carried out the analysis, FNW, SA, UA wrote the manuscript. All authors read and approved the final version of the manuscript.
